# Orthopaedic challenges for mucopolysaccharidoses

**DOI:** 10.1186/s13052-018-0557-y

**Published:** 2018-11-16

**Authors:** Andrea Borgo, Andrea Cossio, Denise Gallone, Francesca Vittoria, Marco Carbone

**Affiliations:** 1 Orthopaedic Clinic, Padua University General Hospital, Padua, Italy; 2 Department of Traumatology and Orthopaedic Surgery, San Gerardo Hospital Milano Bicocca Medical School, Monza, Italy; 3 Institute for Maternal and Child Health IRCCS Burlo Garofolo, Via dell’Istria, 65, 34137 Trieste, Italy

## Abstract

Mucopolysaccharidoses (MPS) are a group of diseases characterized by abnormal accumulation of glycosaminoglycans (GAGs). Although there are differences among the various disease types, the osteoarticular system is always involved. The aim of the present study was to establish a framework for MPS-related orthopaedic manifestations and for their treatment. The authors, affiliated to three different Italian Orthopaedic Centres, report data taken from the literature reviewed in light of their accumulated professional experience. Bone alterations make up what is known as dysostosis multiplex, involving the trunk and limbs and with typical radiological findings. Joints are affected by pathological tissue infiltrations. The cervical spinal cord is involved, with stenosis and cervical and occipitocervical instability. In MPS there is a much higher incidence of scoliosis compared with healthy subjects without any particular distinctive feature. Kyphosis of the spine is more frequent and also more severe because of its possible neurological complications, and it is localized at the thoracolumbar level with a malformed vertebra at the top of the deformity. Evolving forms, and those associated with neurological damage, require anteroposterior spine fusion. The hip is invariably involved, with dysplasia affecting the femoral neck (coxa valga), the femoral epiphysis (loss of sphericity, osteonecrosis), and the femoral acetabulum which is flared. All these features explain the tendency to progressive hip migration. Genu valgum is often found (a deviation of the physiological axis with an obtuse angle opening laterally). This deformity is often localized at the proximal tibial metaphysis; it causes functional limitations and leads to an irregular erosion of the articular cartilage. In young patients who still have the growth plate, it is possible to execute a medial hemiepiphysiodesis, a temporary inhibition of cartilage growth, with progressive axis correction. In this paper, the characterisation of clinical features and the review of treatments are divided into separate sections based on the part of the body involved. The conclusions of each section are presented as a summary. One section discusses the high risk of anaesthesia-related complications requiring the collaboration of specifically trained personnel.

## Background

Mucopolysaccharidoses (MPS) are a group of diseases characterized by abnormal accumulation of glycosaminoglycans (GAGs) in tissues. Although recent approaches, such as enzyme replacement therapy (ERT) and haematopoietic stem cell transplantation (HSCT), have partially improved the orthopaedic conditions, the osteoarticular system is invariably involved and thus the first specialist consulted is often the orthopaedic specialist.

Even though the pathogenesis of the disease is still not fully understood, it seems that GAG deposition in the connective tissue may play a key role in the osteoarticular manifestations through alterations in ossification and chronic inflammation.

Some general features are shared by the different types of MPS, primary amongst which is joint stiffness due to deposition of GAGs during childhood with consequent thickening of capsules and ligaments. This is associated later on, as the disease progresses, with discrepancies in the deformed articular surfaces.

Stiffness is typically localized to the hands and shoulders, but it can affect all joints. This is a common feature of all MPS, except MPS IV where, on the contrary, there is joint hypermobility. Stiffness and articular deformity might require differential diagnosis with rheumatic diseases, but in MPS there are neither clinical nor humoral signs of inflammation [1].

The authors of the present paper are orthopaedic specialists with specific expertise in the field of paediatric pathology. The aim of the study was to review MPS-related orthopaedic manifestations, discussing concepts drawn from professional experience analysed in the light of recent scientific literature on the topic.

### General features: The dysostosis multiplex

Radiologically, the skeletal deformities are labelled as dysostosis multiplex, characterized by severe abnormalities in the development of skeletal cartilage and bone. They consist of anomalies of the skull (macrocephaly, skull thinning), trunk (broadening of the clavicles, oar shaped ribs), spine (odontoid hypoplasia, thoracolumbar vertebral hypoplasia with angular kyphosis), limbs (short and wide diaphysis, irregular metaphysis, flattened epiphysis), hands (bullet-shaped phalanges), pelvis (flared iliac wings, flattened acetabulum, coxa valga, dysplastic femoral epiphysis), and knee (genu valgum) [2].

Short stature, which is a marker of inadequate growth, may be more or less pronounced [3]. In MPS I, IV, and VI, short stature is evident from infancy. In MPS II, stature can be higher, compared with average, up to the age of 8 years, after which growth velocity decreases progressively, finally resulting in short stature. In MPS III, stature is normal or close to normal. Furthermore, in MPS I, IV, and VI, the short stature is disharmonic, with a reduced trunk-to-limb ratio.

Because of impaired skeletal growth, delayed ossification and skeletal age are evident in radiological images.

In summary, in MPS patients the osteoarticular system is invariably involved with different degrees of severity depending on the type and stage of the disease.

### Spine: Kyphosis and scoliosis in MPS

Spine involvement is common in the majority of MPS, although with great variability [4]. Flattened vertebral bodies (platyspondyly) are a typical feature of dysostosis. Wedge deformity of the vertebrae at the thoracolumbar junction (in MPS I, II, IV, and VI) can have dangerous consequences and shares some similarities with congenital kyphosis due to failure of formation. In fact, there exists a congenital defect in vertebral body formation at the thoracolumbar level that leads to progressive development of kyphosis in early infancy. The angular thoracolumbar kyphosis may progress, causing neurological damage as a result of the compression of the contents of the vertebral canal, with consequent ischaemic damage [2]. Standing up becomes more difficult because of forward misalignment of the trunk. Radiological evaluation of the vertebral deformity in anteroposterior and lateral views is performed with patients standing or sitting, measuring Cobb’s angle (the angle between the upper and lower end vertebrae) for kyphotic and scoliotic (if present) assessment.

Magnetic resonance imaging (MRI) may help to identify preclinical signs of spinal cord involvement and localize them to the occipitocervical, cervical, or thoracolumbar regions. The role of somatosensory evoked potential (SSEP) and motor evoked potential (MEP) registration in medullary compression diagnosis is, as yet, not fully established [5].

**Fig. 1:**
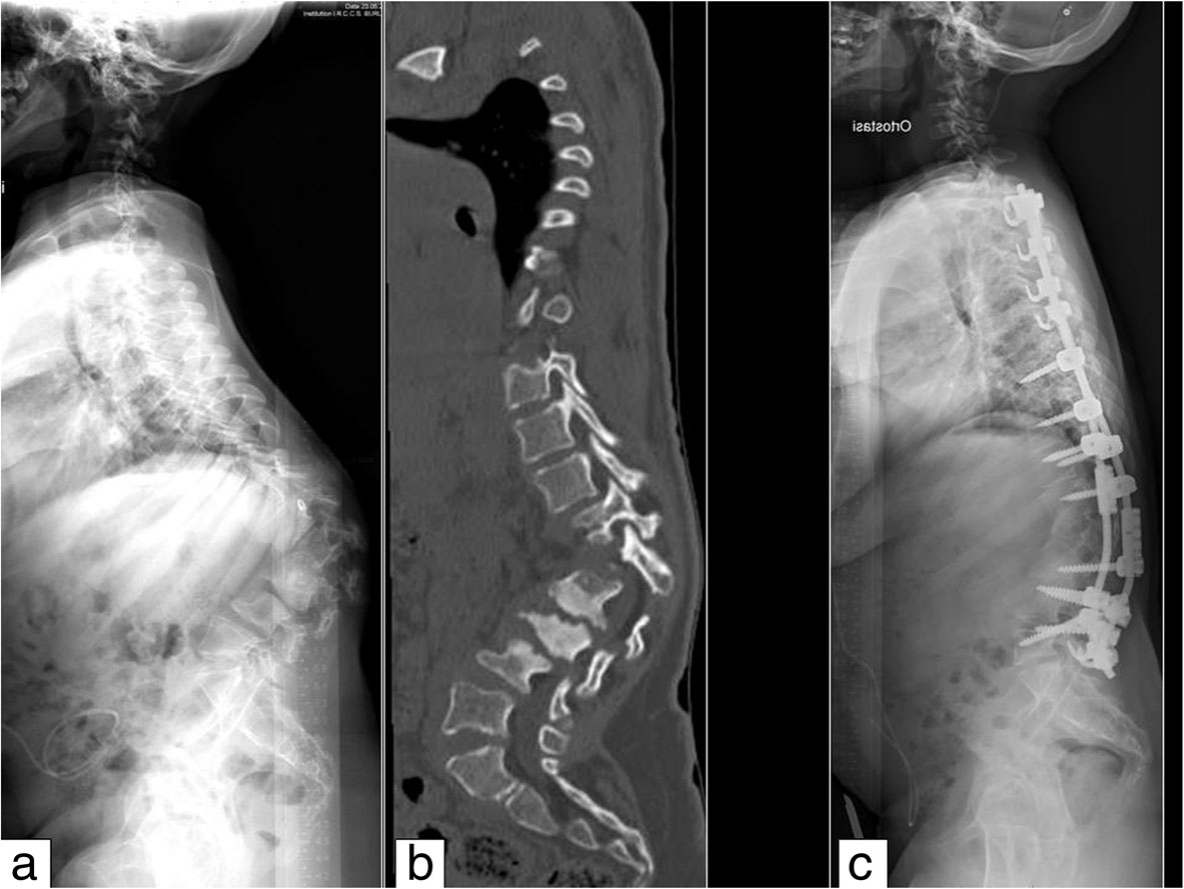
Thoracolumbar kyphosis in a 12-year-old girl with MPS I. **a** Preoperative lateral x-ray showing severe wedge deformity of L1, with kyphosis of about 90°. **b** Computed tomography scan shows the dysostosis of the vertebrae. **c** Postoperative lateral x-ray showing good correction of the kyphosis after anterior fusion with tricortical bone graft and instrumented posterior fusion

Orthopaedic treatment by means of bracing can improve stability and deambulation, but its influence on the evolution of deformities is not supported by clinical evidence. Once the deformity angle progresses beyond 40 degrees of amplitude it tends to aggravate, requiring a surgical approach [6], especially in MPS I and VI. Cases reported in the literature suggest that surgical treatment should be performed before adolescence, on average between the ages of 3 and 8 years [2, 7, 8]. Like all cases of kyphosis, whether congenital or in a skeletal dysplasia setting, angular kyphosis also requires a circumferential arthrodesis, which uses both an anterior and a posterior approach (Fig. 1). The literature suggests a posterior decompression associated with instrumentation and fusion in MPS IV [5]. Anterior fusion entails malformed vertebra removal and replacement with a bone graft from a tissue bank, preferably with the mechanical support of a mesh (there are different kinds of mesh with different forms and materials, mainly titanium and carbon). Posterior spine arthrodesis requires instrumentation (screws and metal bars) associated with bone grafts [7].

Despite the lack of consensus in the literature, in the case of mild deformities an isolated posterior vertebral arthrodesis can be performed to stabilise the surgical correction in association with periodic post-surgery controls [9].

A surgical approach to these deformities has a high risk of neurological complications that cannot always be predicted or avoided, even with modern electrophysiological monitoring techniques and with regular execution of preoperative MRI of the whole spinal cord. Considering that patients with MPS frequently have cervical and cervico-occipital malformations, in these cases it is not possible to use Halo traction, which is commonly employed in other paediatric pathologies. When the thoracolumbar kyphosis has progressive features, it is fundamental to opt for immediate surgery to avoid the development of major angular deformities which require longer operation times and more extensive corrections, thereby increasing the risk of neurological complications.

MPS IV patients often have kyphosis and cervicothoracic stenosis, which can cause neurological symptoms and require a surgical approach consisting of decompression and spinal fusion [10].

Scoliosis is not common in MPS and, when present, is usually associated with kyphosis and treated in conjunction with the management of sagittal plane deformities. Generally, scoliosis treatment is problematic because of trunk shortness and deformity in MPS, which makes it difficult to design a suitable orthopaedic brace. MPS III is the only type that presents different scenarios of spinal involvement; the cervical spine is usually not affected and the costovertebral malformations typical of MPS are in a milder form. A wide-angle scoliosis may develop, and the surgical management must be tailored to the patient based on the prognosis.

In summary, spine involvement is common in MPS. Thoracolumbar kyphosis can cause neurological damage and may require early surgical treatment with anterior and posterior spine fusion.

### Upper limbs: Joint stiffness, trigger fingers, carpal tunnel syndrome

Stiffness is the main feature of upper limb involvement in MPS and, in the worst cases, may develop during infancy, with a symmetric distribution and a progressive course. Shoulders are involved, with limited adduction. Elbows are limited in flexion-extension and especially pronosupination; this can be one of the first clinical signs of MPS, even in the milder forms. Hands are wide and thick, with ulnar deviation of the wrist. Metacarpophalangeal joints are fixed in extension, and interphalangeal joints (proximal and distal) are fixed in flexion, resulting in the so-called ‘claw hand’.

The motion of the hand is limited by the infiltration of GAGs into the joint capsule, while the same kind of infiltration in the tendon sheath can cause stenosing tenosynovitis (trigger fingers). Trigger fingers at a paediatric age may be congenital when limited to the first finger; if other fingers are affected, an MPS diagnosis should be considered and examined in depth [11]. The treatment of trigger finger can be sometimes surgical, sometimes requiring not only flexor tendon pulley resection but also thorough cleaning of the tendon sheath with removal of GAG-infiltrated peritenonium.

Carpal tunnel syndrome is rare at a paediatric age, while it is present with higher prevalence in MPS I, II, and VI [11]. This manifestation is again linked to pathological tissue infiltration of the carpal tunnel, a narrow anatomical structure on the palm side of the wrist the walls of which are formed by carpal bones and transverse carpal ligament; the tunnel contains the flexor tendons of fingers and the median nerve [12]. Impairment of the latter can be assessed by clinical (thumb opposition impairment) and electromyographic examination, while the typical algic symptomatology is completely silent (burning pain in the first four fingers, especially felt at night). Surgical treatment of this syndrome, which is technically very easy, requires opening of the transverse ligament and removal of the pathological tissue from the tendon sheaths.

In summary, trigger fingers and carpal tunnel syndrome at a paediatric age must raise the suspicion of MPS.

### Hip involvement in MPS and possible indications for surgery

Hip dysplasia is a common feature of MPS; it can cause gait impairment in children [13], leading to severe disability in young adults [14, 15].

MPS-related hip dysplasia varies depending on the type of MPS and can show different degrees of severity. The progression of hip dysplasia in children and adolescents with type I MPS-H (Hurler syndrome) after successful HSCT is well described in the literature with particular reference to poorly developed and vertical acetabulum, increased femoral neck-shaft angle, and gradual deformation of the femoral head. It has been suggested that these characteristics may contribute to hip instability with a consequent tendency to lateral and proximal migration. However, there is no consensus on the percentage and on the extent of the migration itself [14, 16–22]. Recently, a progressive increment of the anterior pelvic tilt has been reported [19], highlighting another important aspect of the anatomy and kinematics of the pelvis and the thoracolumbar spine in MPS I patients. Based on the progression of these anatomical alterations, a surgical reconstruction of the hip can be indicated in MPS I with a combination of both pelvic and femoral osteotomies to restore the normal anatomy of the hip [2, 16, 22, 23]. Hip reconstruction surgery performed in MPS I children can provide a stable, well-covered hip, but appears to be unable to prevent radiological deterioration and clinically significant hip arthritis in the long term. Thus, a total hip replacement may be needed in young adults [24].

Hip morphology and progression of hip alterations in MPS II are similar to that described for MPS I but to a milder extent. Similar to MPS I, there is no consensus regarding the incidence and extent of hip migration in MPS II [17, 18, 25]. Reviewing the literature on MPS I and II, it is clear that there is no strict correlation between the radiological parameters of hip dysplasia and the degree of pain and mobility impairment; some MPS patients may experience hip discomfort with a minor degree of radiographic dysplasia [18]. Furthermore, in MPS I patients there seems to be no correlation between radiographic parameters and clinical characteristics, such as age at the time of transplant (HSCT), ERT pre-HSCT, donor chimerism, and alpha-l-iduronidase activity post-HSCT [19].

Hip involvement in MPS III is less documented in the literature, but appears to be present in almost 50% of patients. Unlike the other forms of MPS, in MPS III wide deformities of the acetabulum are not common; the prevalent features are femoral head deformities and an increased femoral neck-shaft angle, which are rarely noticed before early adolescence [26, 27]. Therefore, MPS III patients may also experience hip pain. Generally, hip involvement in MPS III starts to be clinically relevant when the general conditions of these patients start to deteriorate. It is mostly treated with symptomatic therapy and steroid injections. Only rarely are major surgical treatments (femoral head removal, total hip arthroplasty) reported in the literature [23, 26, 27].

In MPS IVA, the hip shows anatomic alterations similar to MPS I but with a reduced tendency to lateral migration, especially during early infancy. In adolescents and in young adults, the main problems are the deformity of the femoral head and the poor development of the acetabulum [28, 29]. Surgical hip management in infants with MPS IV is equivocal in the literature; some cases of hip reconstruction during infancy are reported, but there is a tendency to partial relapse of the migration. In adults, total hip arthroplasty should be considered [23].

In MPS VI, the progressive deformity of the femoral head as well as a lateral migration of the hip is already detectable in early infancy. Usually the migration is only partial and tends to be stable over time; pain and impairment are absent during the growing age. The literature on hip management in MPS VI during infancy is poor and the role of surgery is not clear while, in young adults, total hip replacement should be considered [2, 30].

In summary, hip dysplasia is a common finding in MPS. In MPS children, the hip should be followed-up with plain x-rays. During the growing stage, surgical hip reconstruction can be considered, while in young adults an indication for total hip arthroplasty can be given. Indications for surgery are mostly based on the progression of the disease and on the symptoms.

### Lower limbs: What are the current indications for surgical treatment of genu valgum in MPS?

In MPS patients a valgus deformity of the knee and ankle are often present, and a valgus deformity of the foot is also commonly observed, with the knee being the most frequently affected lower limb joint [31]. Valgus deformity is typically progressive from when ambulation starts and, in most cases, becomes severe enough to make surgery necessary. The deformity is mostly due to the proximal tibia, but the distal femur can also contribute. Progressive collapse and fragmentation of the tibial and femoral epiphysis becomes evident at radiographic examination [29].

The ankles of these patients are typically in valgus, with wedging of the distal tibial epiphysis and shortening of the fibula. In addition, valgus hindfoot can be detected, with some degree of equinus, and forefoot adduction, with some degree of supination and prominence of the fifth metatarsal head dorsally. In lateral x-rays, the tarsal bones appear small and irregular with plantarflexion of the talus.

Considering the progression of the deformity, close clinical follow-up is important to ensure timely intervention to prevent disability. Clinical evaluations should include assessment of lower limb alignment, range of motion (ROM) of the joints, and movement patterns. The knee may show a flexion contracture. In case of genu valgum, a good clinical method to assess deformity is measurement of the intermalleolar distance (in orthostatic and supine position). Of crucial importance is the evaluation of ligamentous laxity (with valgus varus stress tests) which can aggravate the deformity and is a distinctive element of MPS IV.

Adequate clinical evaluation must be accompanied by the radiographic study of the pelvis and standing, full-length lower limbs in anteroposterior view to assess the mechanical axis measurements [32], and knee and ankle measurements (in particular the tibio-femoral angle, lateral distal femoral angle, and medial tibial proximal angle) [33]. Radiographic follow-up is essential in patients who show progression of the deformity at clinical evaluation.

**Fig. 2:**
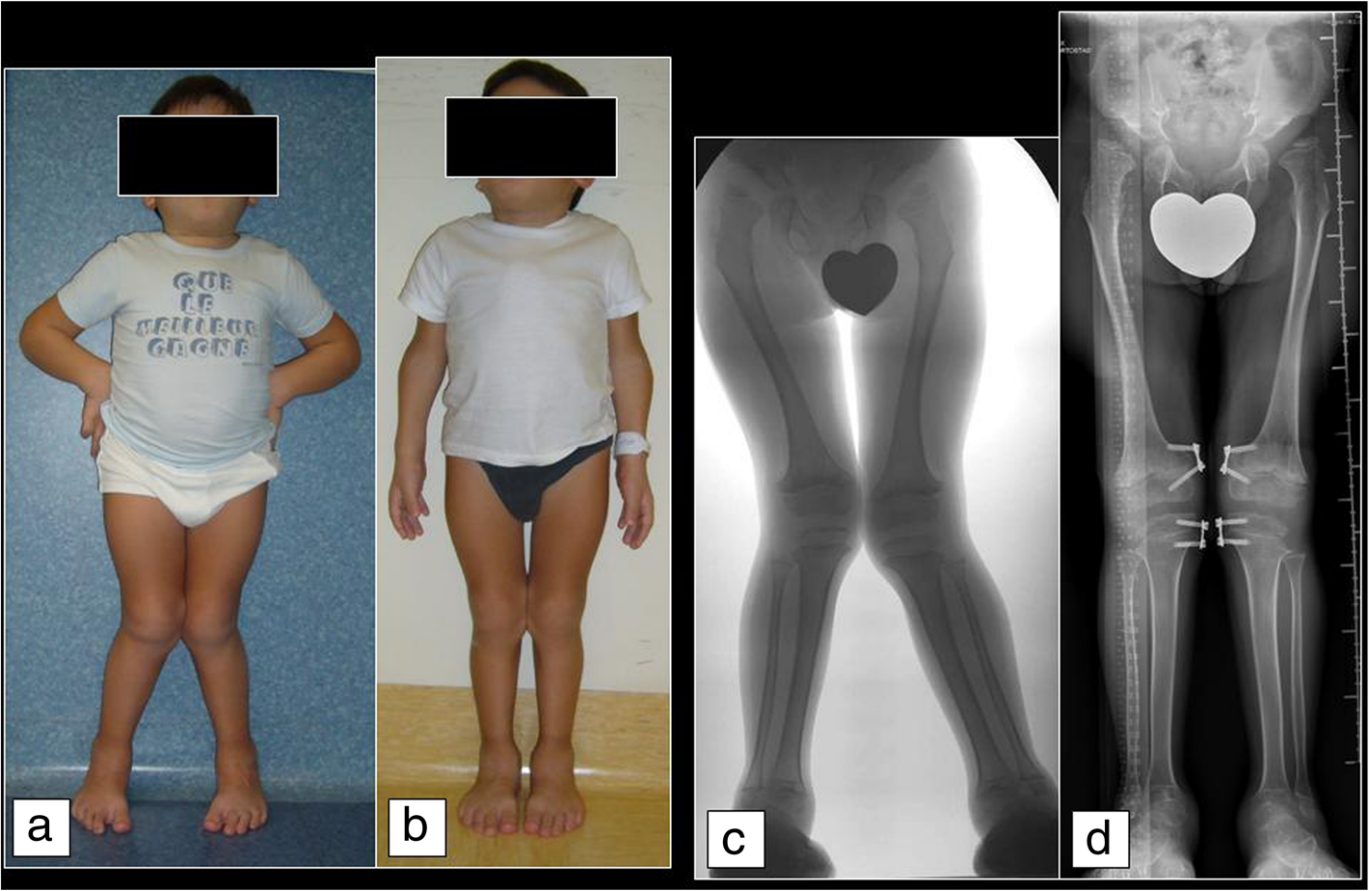
Genu valgum in a 9-year-old boy with Morquio disease (MPS IVA). **a** Before temporary bilateral hemiepiphysiodesis of femur and tibia with Eight Plates. **b** After 2 years and 8 months, showing good correction of the valgus deformity. Clinical appearance of the patient **c** before surgery and **d** before plate removal

In general, there are no definitive guidelines for the treatment of skeletal deformity in lower limbs in patients with MPS. For instance, the knee is considered pathological when the femoral-tibial angle is greater than 7°; however, the indication for surgical treatment should be given when it reaches 15° of deformity [2, 34]. Regarding treatment options, hemiepiphysiodesis techniques (the so-called guided-growth techniques) have been shown to be simple and effective in reducing the deformity. However, since the growth potential is limited, correction is likely to take a long time. The guided-growth principle is applied through temporary load modulation of the growth of the physis. In the past this was achieved using Blount staples, nowadays with the so-called tension band plate [34, 35]. It may be necessary to leave the devices in place for 2–3 years or more to obtain a good correction, but this does not seem to be associated with damage or closure of the physis (Fig. 2). Taking the characteristics of these patients into account, treatment should be started as soon as deformity becomes evident, even as early as 4 years old. Postoperative hospitalisation may be indicated depending on the complexity of the patient’s condition; however, the procedure can usually be carried out as an outpatient [29, 35].

In cases of severe deformities associated with low growth potential, or in young adults with closure of the physis, osteotomy is recommended to obtain proper correction. Proximal tibial correction may be performed acutely through an oblique osteotomy, or gradually with an external fixator. For severe femoral valgus deformity, distal femoral valgus osteotomy and correction with an external fixator is the best option as it allows for ideal correction of the mechanical axis despite there being a high incidence of deformity recurrence after correction, mostly in the proximal tibia [36]. It is important to remember that osteotomies are major surgical interventions characterised by high complication rates and a long recovery time after surgery. In patients who are at high risk of losing the walking ability they had prior to surgery, these factors can lead to an impossibility of regaining preoperative function and, in some case, permanent loss of independent mobility [35]. The worsening of the deformity can lead to the development of symptomatic osteoarthritis requiring prosthetic replacement. There are only a few reports on this subject in the literature; however, they seem to indicate that these patients tend to require custom-made implants [37, 38].

Skeletal deformities are a characteristic feature of MPS and can be the cause of major disability. Those in the lower limbs often require repeated surgical interventions which should be carried out as early as possible, even at the age of 4 years. In patients with major deformities, or those who are older, correction with hemiepiphysiodesis may be required even in the distal femur [35]. In these cases, it is important to attain some degree of overcorrection since there is a high percentage of recurrence of the deformity [21]. Treatment with guided growth procedures has proven to be effective in correcting lower limb defomities and increasing joint ROM by up to 30% [35].

Radiographic studies and gait analysis suggest that the deformities in the ankle and foot are primarily due to eversion of the subtalar and inactivity/weakness of the tibial posterior, both of which may be exacerbated by the valgus knee. In the majority of cases, foot and ankle problems can be treated with orthesis. In patients with good growth potential, the indication, also in the case of valgus ankle, is to perform a tibial hemiepiphysiodesis, while osteotomy is indicated for greater deformities in older patients, bearing in mind that, despite treatment, the deformity may recur [36].

In summary, temporary hemiepiphysiodesis with tension-band plates is an adequate solution for MPS patients with genu valgum, provided the growth potential is good.

### To remember: Complications during anaesthesia

When giving indications for surgical intervention, the orthopaedic surgeon must take into due account the increased risks associated with general anaesthesia. This is especially true for patients affected by MPS as a result of reduced mouth opening, macroglossia, and frequent short and stiff cervical spine. The latter can be associated with cervical instability and stenosis (MPS IV and VI), which requires extra caution when performing intubation manoeuvres [2]. These patients should only be managed by anaesthesiologists who have experience in this field and with the aid of a radiological examination performed within the 12 months preceding the surgery. An emergency tracheotomy may become necessary at any moment [39].

In MPS II, GAG infiltration of the whole respiratory tract causes tracheal stenosis and may lead to severe complications that must be considered when general anaesthesia is planned [40].

In MPS I and IV, thoracolumbar kyphosis (possibly associated with cervicothoracic kyphosis in MPS IV) can cause neurological lesions with paraplegia; these may also occur in extraspinal surgical corrections, such as corrective osteotomy for hip dysplasia and osteotomy of the femur and tibia to correct knee valgus [41], even though the pathogenesis is not yet clear.

## Conclusions

In summary, MPS patients are at high risk of neurological, anaesthesiological, and general surgical complications.

## Abbreviations

ERT: Enzyme replacement therapy
GAG: Glycosaminoglycan
HSCT: Haematopoietic stem cell transplantation
MPS: Mucopolysaccharidosi(e)s
MRI: Magnetic resonance imaging
ROM: Range of motion
